# Primary neuroendocrine tumors of the breast: two case reports and review of the literature

**DOI:** 10.1186/s13256-020-02361-5

**Published:** 2020-03-10

**Authors:** Loubna Hejjane, Karima Oualla, Zineb Bouchbika, Mouna Bourhafour, Anas Lhlou Mimi, Efared Boubacar, Abdellatif Benider, Zineb Benbrahim, Samia Aarifi, Nawef Mellas

**Affiliations:** 1 Mohammed VI Center for Cancer Treatment, Ibn Rochd University Hospital, Casablanca, Morocco; 2 Department of Oncology Medical, Hassan II University Hospital, Fez, Morocco; 3 Department of Radiology, Hassan II University Hospital, Fez, Morocco; 4 Department of Pathology, Hassan II University Hospital, Fez, Morocco

**Keywords:** Neuroendocrine carcinoma, Breast cancer, Case report, Chromogranin, Immunohistochemistry

## Abstract

**Background:**

Neuroendocrine carcinomas mainly affect the bronchopulmonary and the gastrointestinal systems. Breast localizations are very rare. They represent less than 0.1% of all breast cancers. A definitive diagnosis relies on histological and immunohistochemical examinations.

**Case presentation:**

Case 1

We report a case of primary neuroendocrine carcinoma of the breast in a 71-year-old Arabic woman who presented with a 3 cm palpable and mobile tumor of the right breast. Clinical and radiological assessment excluded any other primary tumor. Radical mastectomy and axillary lymph node resection were performed. A histopathological examination disclosed the diagnosis of primary breast neuroendocrine tumors, with negative surgical margins and lymph nodes (18 N-/18 N). The tumor cells were positive for neuroendocrine markers, a weak Ki-67 proliferation index and negative Her2/neu. Our patient received adjuvant hormonal treatment with anti-aromatase for 21 months. She is on regular follow-up, and she remains free of disease to date.

Case 2

A 48-year-old Arabic woman consulted for a right breast nodule. She underwent lumpectomy with right axillary lymphadenectomy. The diagnosis was breast neuroendocrine tumor. Systemic treatment was proposed, but she was lost to follow-up. She consulted 1 year later for a mass in the same breast. A histological and immunohistochemical examination of a mammary biopsy was consistent with a recurrence of the previous neuroendocrine tumor. A radiological assessment showed a large mass in her right breast, ipsilateral axillary lymphadenopathies, and hepatic and pulmonary metastases. She received first-line metastatic chemotherapy, with good clinical and radiological improvement. She refused the mastectomy and was given hormone therapy. One year later, the tumor expanded clinically and radiologically, and she underwent second-line metastatic chemotherapy, with good clinical progress and radiological stability, and she then underwent maintenance hormonal therapy.

**Conclusion:**

Due to the rarity of primary breast neuroendocrine tumors, no standard therapy exists and the prognosis remains difficult to determine. Studies, including larger series, are needed in order to understand the biological behavior of these tumors.

## Background

Primary breast neuroendocrine tumors are a rare histological type representing less than 0.1% of all breast cancers [1, 2]. These tumors were initially described by Cubilla *et al.* [3] in 1977; since then, additional cases have been reported. Primary neuroendocrine carcinomas of the breast are currently included in the latest World Health Organization (WHO) classification of breast tumors [4]. We report two cases of primary breast neuroendocrine tumors, with a literature review.

## Case presentation

### Case 1

**Fig. 1:**
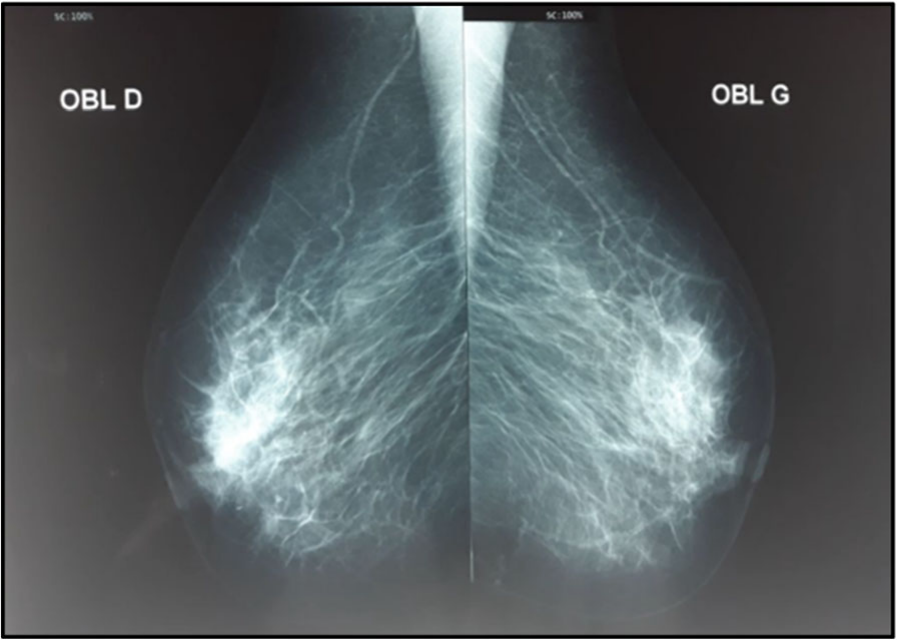
Mammographic profile showing an opacity above-nipple in the right breast

**Fig. 2:**
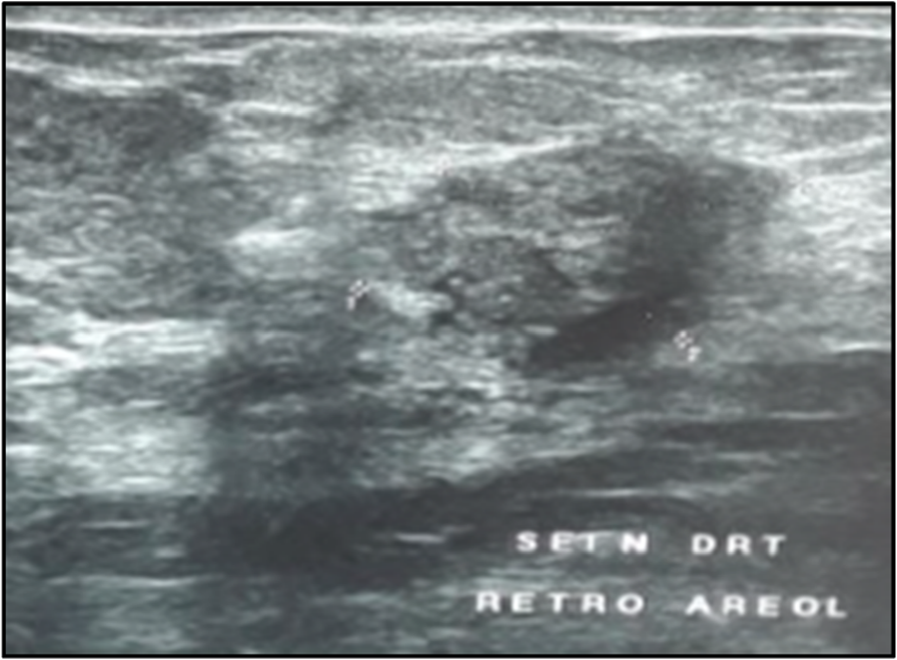
Ultrasound appearance showing above-nipple tissue mass in the right breast, measuring 18 × 14 mm, with irregular hypoechoic contours; American College of Radiology 4 classified lesion

A 71-year-old menopaused Arabic woman, with no medical history presented with a 3 cm right breast nodule that gradually increased size during 1 year. At a physical examination the nodule was located at the upper quadrants junction, with no inflammatory signs, and her axillary lymph nodes were not palpable. The rest of the physical examination was normal. Mammography (Fig. 1) and a breast ultrasound (Fig. 2) showed an 18 × 14 mm hypoechoic nodule above the nipple with irregular contours, assessed as Breast Imaging Reporting and Data System (BIRADS) 4c. A biopsy of the nodule was performed and disclosed the diagnosis of an invasive breast carcinoma of no special type, graded as Scarff–Bloom–Richardson (SBR) II.

**Fig. 3:**
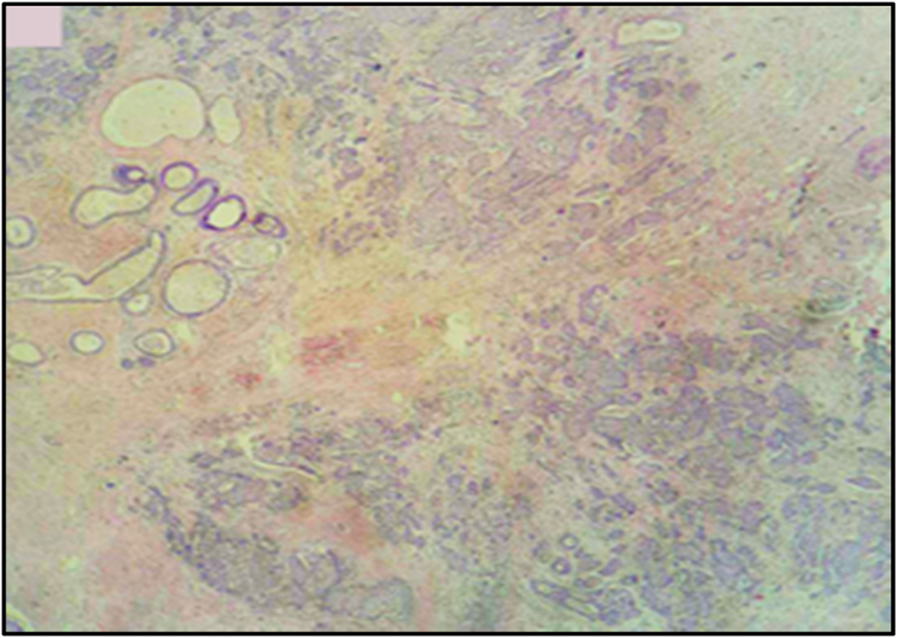
Histological aspect showing a breast parenchyma with tumor proliferation in small masses of variable size with a fibrous stroma (hematoxylin, erythrosine, and saffron × 100)

**Fig. 4:**
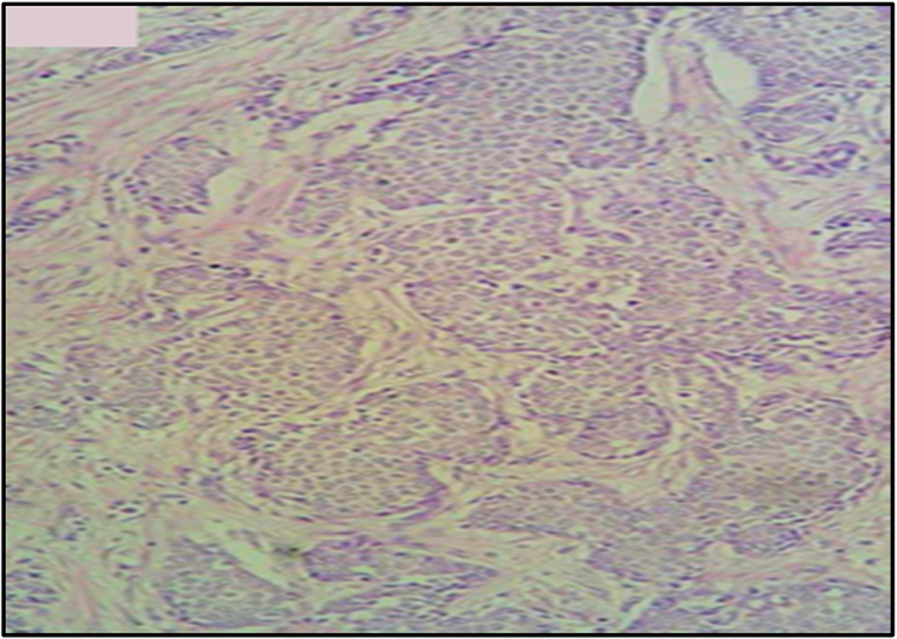
Histological appearance showing monomorphic tumor cells, a little atypical with rounded nuclei and scant cytoplasm (hematoxylin, erythrosine, and saffron × 200)

A thoracoabdominal computed tomography (CT) scan and bone scintigraphy imaging ruled out any other primary disease. Radical mastectomy and axillary lymph node resection were performed. A histopathological examination of the resected specimen showed a 2.2 cm tumor consisting of an invasive malignant tumor suggesting neuroendocrine carcinoma, with 5% intratumoral cribriform ductal carcinoma *in situ* with intermediate nuclear grade (Figs. 3 and 4). The surgical margins were negative as well as the 18 axillary lymph nodes (18 N-/18 N).

**Fig. 5:**
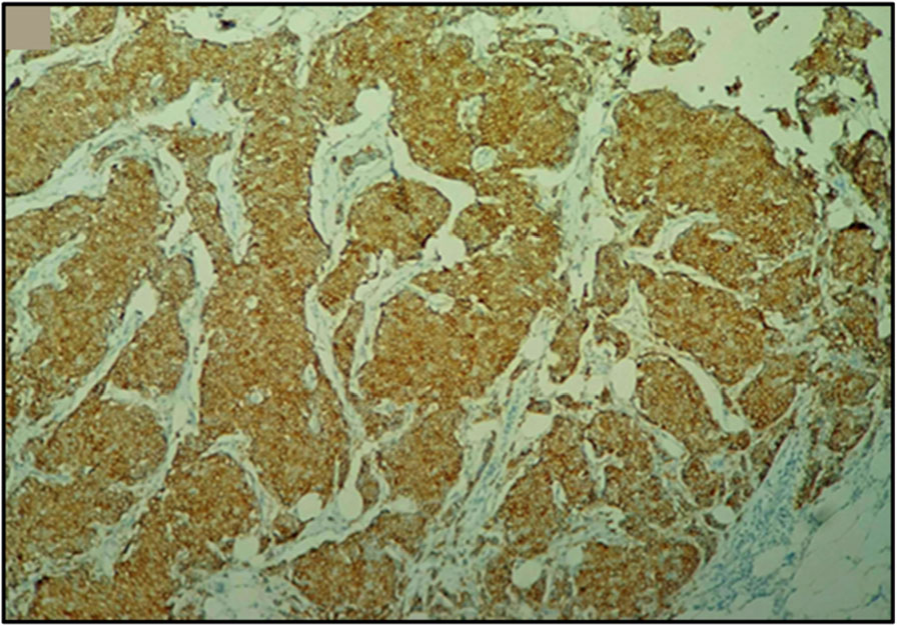
Immunohistochemical aspect showing massive expression at synaptophysin (× 100)

**Fig. 6:**
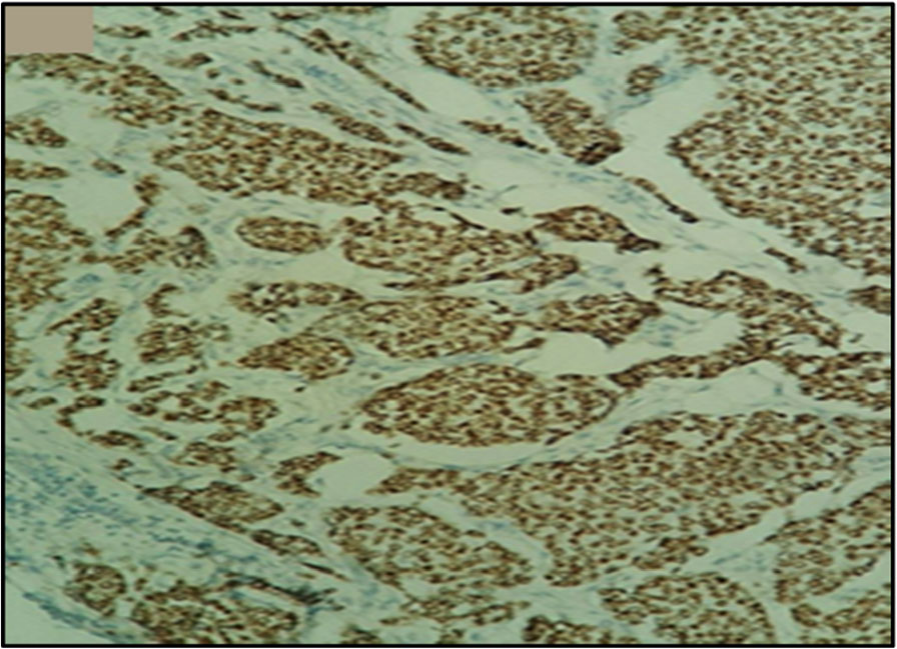
Immunohistochemical aspect showing an expression of estrogen receptors (× 100)

At immunohistochemical analysis, tumor cells stained positive with synaptophysin (Fig. 5), chromogranin, cytokeratin, and neuron-specific enolase (NSE). Hormonal receptors were highly expressed (Fig. 6) (estrogen receptors, 90%; progesterone receptors, 90%), the Ki-67 proliferation index was 5% and Her2/neu was negative. The definitive diagnosis of our patient was primary breast neuroendocrine carcinoma, and she subsequently received adjuvant hormonotherapy with anti-aromatase (letrozole 2.5 mg daily) for 21 months. She is on regular follow-up at our cancer center, and she is free of disease to date.

### Case 2

A 48-year-old Arabic woman with unremarkable medical history presented with progressively enlarging nodule of 2 years’ duration. A mammography showed an opaque heterogeneous mass with irregular contours between the right breast lower quadrants measuring 23 mm.

Ultrasound scanning showed a hypoechoic solid formation, with irregular contours, measuring 21 × 16 mm, enclosing a rich Doppler signal, very suspicious of malignancy, graded as BIRADS 5.

**Fig. 7:**
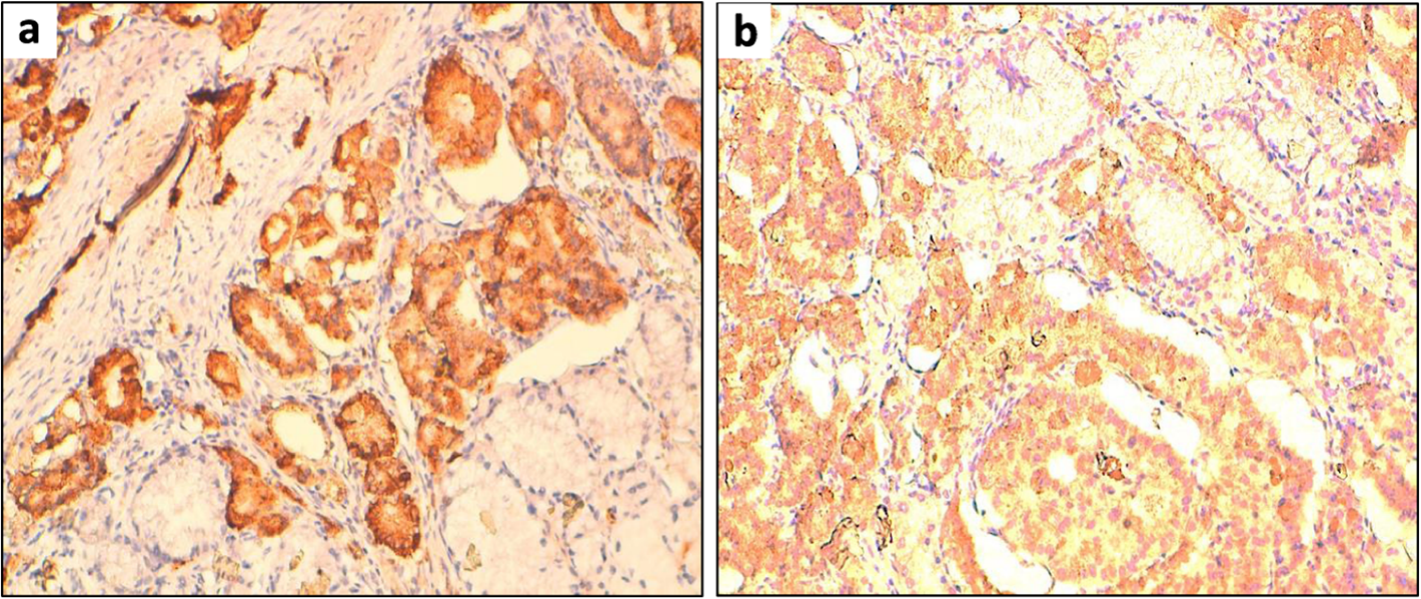
**a** Immunohistochemistry showed synaptophysin positivity in the tumor cells (× 100 magnification). **b** Immunohistochemistry showed chromogranin positivity in the tumor cells (× 200 magnification)

An ultrasound-guided microbiopsy of the right breast nodule was performed. Histopathological analysis disclosed the diagnosis of a neuroendocrine carcinoma of the right breast, with a significant positive immunostaining for chromogranin and synaptophysin by tumor cells (Fig. 7).

A surgical lumpectomy and right axillary dissection were performed. Macroscopic examination of the resected specimen showed a 4 × 2 × 2 cm whitish nodular tumor with a hard consistency. On microscopic examination, it was a malignant invasive breast neoplasm suggesting neuroendocrine carcinoma, SBR grade III, with endovascular emboli and negative surgical margins. There were seven metastatic lymph nodes with capsular invasion and endolymphatic emboli (7 N +/17 N). An immunohistochemical analysis of the tumor showed positive staining for synaptophysin and chromogranin antibodies.

Systemic treatment was prescribed for our patient; however, she was lost to follow-up. She came back 12 months later for mastodynia in the same breast with hardening of the retro-areolar part and nipple retraction. A clinical examination showed a 13 cm mass in her right breast at the level of the infero-external quadrant, which was hard, painful, and fixed to the superficial breast tissues with nipple retraction, associated with two large ipsilateral axillary lymphadenopathies measuring 10 and 6 cm. A histopathological analysis of the tumor biopsy was consistent with a recurrence of her previous SBR grade III neuroendocrine carcinoma. Hormonal receptors were positive (estrogen receptors, 80%; progesterone receptors, 1%). The Ki-67 proliferative index was 60% with negative HER2/neu.

A radiological assessment was performed: bone scintigraphy showed no secondary bone lesion, and a thoraco-abdominopelvic CT scan showed a large right breast mass, right axillary lymphadenopathy, and liver and lung metastases. She received a first-line metastatic chemotherapy with eight cycles of anthracyclines (epirubicin 100 mg/m^2^ and cyclophosphamides 500 mg/m^2^) with a good clinical course and a clear regression of the tumor, which became softened, as well as of the metastatic axillary lymph nodes. An evaluation by thoraco-abdominopelvic CT scan showed a regression of the pulmonary and hepatic nodules after she was prescribed tamoxifen maintenance hormone therapy 20 mg/day.

After 4 months, the clinical evolution was satisfactory: the tumor and the axillary lymphadenopathy were no longer palpable. At a thoraco-abdominopelvic CT scan, liver metastases had decreased in size and the pulmonary nodules had disappeared. A multidisciplinary meeting suggested a mastectomy, but our patient refused; thus, she was kept on tamoxifen hormone therapy 20 mg/day.

One year later, the tumor progressed clinically and radiologically with a reappearance of a 6 cm hemorrhagic fistulized mass in her right breast, with nipple infiltration, and a hard fixed axillary lymphadenopathy.

Bone scintigraphy did not show secondary bone lesions. A thoraco-abdominopelvic CT scan showed multiple right breast masses associated with subcutaneous thickening and bilateral infracentimetric axillary adenopathies, and multiple nodular hepatic lesions, the largest measuring 22 × 13 mm. Biological assessment revealed a high level of CA 15-3 up to 213.2 U/ml.

A second-line metastatic chemotherapy with paclitaxel 175 mg/m^2^ – carboplatin AUC 5 was prescribed for our patient.

After six courses of chemotherapy, there was a good clinical evolution with regression of the breast mass, disappearance of axillary lymphadenopathies, and a decrease level of CA15-3 up to 180 U/ml. The hepatic and pulmonary metastases remained stable.

Our patient then underwent hormonal therapy maintenance with letrozole 2.5 mg/day with a good tolerance.

## Discussion

Neuroendocrine carcinomas of the breast are very uncommon; they are little-known tumors, representing less than 0.1% of all breast cancers and less than 1% of neuroendocrine tumors [1]. Classically described in the pulmonary and digestive systems, they can also be seen in other extrapulmonary and extradigestive locations. Primary mammary gland localization is very rare [1]. Neuroendocrine tumors are mainly observed in white women aged from 60 to 70 years [1], but younger patients have been reported in the literature. Men can also be affected by these tumors [5, 6].

Primary neuroendocrine tumor of the breast is a diagnosis of exclusion. An Octreoscan and a positron emission tomography (PET) scan should rule out primary sites: ears, nose, and throat (ENT), lungs, digestive, and cutaneous [2, 7]. No clinical signs are specific to these tumors [8, 9]. These tumors are characterized by a slow evolution and the most frequent reason for consultation is an isolated breast nodule or a breast nodule associated with other signs [8]. Our cases illustrate these features that are reported in the literature. Sometimes these tumors present as a well-limited erythematous and purplish cutaneous lesion [8].

The 2003 WHO classification of breast tumors recognizes neuroendocrine carcinoma as a distinct histological entity, with the same morphological characteristics as neuroendocrine tumors of the gastrointestinal tract, pancreas, and lung, with more than 50% positive immunostaining of tumor cells by a neuroendocrine marker such as chromogranin or synaptophysin [4].. Four groups are described: solid neuroendocrine carcinomas, atypical carcinoids, small cell carcinomas, and large cell neuroendocrine carcinomas [4]. This classification excludes mammary carcinomas with focal neuroendocrine differentiation defined as scattered tumor cells that stain positive with neuroendocrine markers [10, 11]. Focal neuroendocrine differentiation is reported in approximately 2–5% of breast cancers [12]. Sapino *et al*. proposed a classification of breast endocrine tumors into five types: the cohesive solid variant; the alveolar form; the small cell form; the papillary solid variant; and mucinous carcinoma [13, 14]. These last two forms are characterized by the production of mucus and the frequent association with an *in situ* component with endocrine differentiation [14].

There is no specific clinical or radiological sign to diagnose a neuroendocrine carcinoma; a histological examination is the only way to confirm the diagnosis of this tumor [8, 9]. On macroscopic examination, primary neuroendocrine carcinomas of the breast are round or multilobulated, yellowish-colored, and have a firm consistency, or, rarely, they are gelatinous if associated with a mucinous component [4, 13]. At histological examination, a diagnosis of the neuroendocrine differentiation of these tumors can be suspected morphologically and should always be confirmed after immunohistochemical analysis by showing positive staining with neuroendocrine markers by at least 50% of the tumor cells. Chromogranin and synaptophysin are the most sensitive and specific neuroendocrine markers [14, 15]. In our cases, the two markers were expressed. Other less specific markers can also be expressed: NSE, neuron cell adhesion molecules (NCAM), neurofilament, and bombesin. High molecular weight cytokeratins are negative. The expression of Her2 is generally absent whereas the estrogenic and progesterone receptors are strongly expressed as in our patients [16, 17]. Strict histological criteria have been defined for the diagnosis of primary breast neuroendocrine carcinoma: the presence of an *in situ* component and/or the absence of extramammary localization [1].

According to Günhan-Bilgen et al., the diagnosis of a primary endocrine tumor of the breast in imaging can be suggested if a patient presents with a dense mammary mass, with microlobulated or spiculated contours on mammography, and with a hypoechoic and homogeneous appearance on ultrasound [18]. However, these imaging features are not specific as a hypoechoic and homogeneous appearance on ultrasound can be seen in other malignant tumors (such as grade 3 infiltrating carcinoma or mucinous carcinoma). Neuroendocrine tumors of other organs may metastasize in the breast; their appearance in this case differs from primary breast neuroendocrine tumors. Secondary mammary neuroendocrine tumors have the same imaging appearance as other breast metastases, they present as well-circumscribed nodules without spicules or calcification.

The treatment of neuroendocrine tumors of the breast is mostly surgical. It combines: mastectomy, axillary dissection, and metastasectomy. The indications for chemotherapy and radiotherapy are the same as for other breast cancers. The combination of an anti-aromatase acts on the mammary component. The neuroendocrine component usually escapes within a few months but can be controlled by anthracycline-based chemotherapy [19]. The indications for hormonotherapy and immunotherapy are not codified because their effects remain uncertain [18].

The evolution of neuroendocrine tumors of the breast is slow. Their prognosis depends mainly on the histological grade and the anatomoclinical stage [13, 20, 21]. These tumors are histologically graded as their counterparts in other sites [4, 13]. Thus, the solid variant of neuroendocrine carcinomas and atypical carcinoids have better prognoses than small cell neuroendocrine carcinomas and undifferentiated large cell carcinomas. The presence of an associated mucinous component would be a factor of a good prognosis [13, 14].

The 5-year survival of breast primary neuroendocrine tumors exceeds 80% in all combined tumor subtypes. However, recent studies specified the frequency of locoregional recurrences and metastases, making the prognosis better in general [8, 14, 22]. The main prognostic factors are the age, the field, the capacity of tumor secretion, the tumor size, and the existence or not of distant metastases [8, 22].

## Conclusion

Neuroendocrine tumors of the breast are rare tumors; they can be primary or secondary. Histopathological analysis is the only way to achieve their correct diagnosis by using appropriate immunohistochemical staining. Studies including larger series are needed in order to understand the biological behavior of breast neuroendocrine tumors.

## Data Availability

All data generated or analyzed during this study are included in this published article.

## Abbreviations

BIRADS: Breast Imaging Reporting and Data System
CT: Computed tomography
ENT: Ears, nose, and throat
NCAM: Neuron cell adhesion molecules
NSE: Neuron-specific enolase
PET: Positron emission tomography
SBR: Scarff–Bloom–Richardson
WHO: World Health Organization
